# Metabolic and life-history effects of heat stress in early life stages of a marine copepod, *Calanus finmarchicus*

**DOI:** 10.1093/plankt/fbag048

**Published:** 2026-06-30

**Authors:** Sidonie E J Rousseau, Justine Hienne, Dag Altin, Kang Nian Yap

**Affiliations:** Department of Biology, Norwegian University of Science and Technology, Høgskoleringen 5, Gløshaugen, 7491, Trondheim, Norway; Department of Biology, Norwegian University of Science and Technology, Høgskoleringen 5, Gløshaugen, 7491, Trondheim, Norway; Research Infrastructure SeaLab, Norwegian University of Science and Technology, Brattørkaia 17C, Brattøra, 7010, Trondheim, Norway; BioTrix, Finn Bergs veg 3, 7022, Trondheim, Norway; Department of Biology, Norwegian University of Science and Technology, Høgskoleringen 5, Gløshaugen, 7491, Trondheim, Norway

**Keywords:** copepod, heat stress, metabolic rate, early life stages, development

## Abstract

Understanding how early life stages of *Calanus finmarchicus* respond to warming is crucial for predicting the resilience of North Atlantic ecosystems under climate change. We experimentally investigated the effects of heat stress on resting metabolic rate and key life-history traits, including hatching success, developmental time, body size and mortality, as well as the combined effects of temperature and food availability on naupliar survival. Increasing temperature (10°C–24°C) accelerated hatching and development but reduced hatching success, with no hatching observed at 23°C and 24°C. Nauplii developed faster at 15°C than at 10°C but were smaller, indicating a trade-off between growth and developmental rate. Mortality increased with temperature (10°C vs 15°C), although food availability partially mitigated this effect, suggesting that energetic input can buffer, but not fully compensate for thermal stress. Egg metabolic rate remained stable across temperatures, while naupliar metabolic rate followed a thermal performance curve, peaking at 15°C and declining at higher temperatures. These findings reveal strong, stage-specific thermal sensitivities and highlight physiological trade-offs between metabolic demand, growth and survival. As marine heatwaves become more frequent, such responses could lead to smaller, less viable individuals and reduced prey quality, ultimately affecting trophic transfer and marine population productivity.

## INTRODUCTION

Climate change is driving a rapid increase in ocean temperatures, particularly in the North Atlantic Ocean, where recent marine heatwaves (2023–2024) have pushed sea surface temperature to ~20°C and higher (Terhaar *et al.*, 2025). The Intergovernmental Panel on Climate Change (IPCC, 2023) projects that such marine heatwaves will become more frequent and intense, with cascading consequences for marine ecosystems (Doney *et al.*, 2012). Temperature strongly regulates physiological and biochemical processes in marine ectotherms, including metabolic rate, development and reproduction (Schulte, 2015). In zooplankton such as copepods, these processes are tightly coupled to ambient temperature, making them particularly vulnerable to thermal variability (Richardson, 2008). Because of their central roles as primary consumers and as a key food source for fish (Lomartire *et al.*, 2021), seabirds (Stempniewicz *et al.*, 2007) and marine mammals, copepods are critical to both marine food webs and carbon cycles (Steinberg and Landry, 2017).

The copepod *Calanus finmarchicus* is one of the most ecologically important species in the North Atlantic Ocean, contributing substantially to zooplankton biomass and energy transfer to higher trophic levels (Grieve *et al.*, 2017). Over the past several decades, and supported by recent studies, *C. finmarchicus* has exhibited climate-driven shifts in distribution, including poleward expansion and increasing occurrence in Arctic regions, alongside changes in life cycle timing associated with ocean warming (Chust *et al.*, 2014; Weydmann *et al.*, 2015; Kristiansen *et al.*, 2021; Tarling *et al.*, 2022). These changes may disrupt the synchrony with phytoplankton blooms and predators, with potential consequences for recruitment success and food web dynamics (Ji *et al.*, 2010).

Temperature is strongly influencing life-history traits in *C. finmarchicus* by enhancing egg production and development rates at low to moderate temperatures (0°C–10°C; Pasternak *et al.*, 2013; Weydmann *et al.*, 2015) but reduces hatching success at higher temperatures (>20°C; Preziosi and Runge, 2014). Numerous studies have demonstrated faster nauplii development at elevated temperatures (Campbell *et al.*, 2001; Cook *et al.*, 2007; Jung-Madsen and Nielsen, 2015) and temperature-dependent changes in body size consistent with the temperature–size rule from Atkinson (1994). However, elevated temperatures can also impose physiological stress, as shown by increased mortality in adults (Rousseau *et al.*, 2025).

*C. finmarchicus’* early life stages (i.e. nauplii) are especially important, as they represent a major food source for fish larvae (Jacobsen *et al.*, 2020). During early development, nauplii rely primarily on endogenous energy reserves prior to the onset of feeding at the N3 stage (Jung-Madsen and Nielsen, 2015), making their survival sensitive to food availability and the timing of phytoplankton blooms. Consequently, understanding how these early stages respond to environmental stressors is essential for predicting ecosystem-level impacts. Despite extensive work on temperature effects on life-history traits (e.g. hatching success, development rate and size), the underlying physiological mechanisms are poorly resolved. In particular, metabolic responses to acute heat stress during early life stages remain largely unexplored in *C. finmarchicus*. Metabolic rate determines how rapidly energy reserves are consumed and thus provides a key link between temperature and organismal performance (Ikeda, 1985). This is especially critical during embryonic and early naupliar stages, when individuals rely on finite energy reserves and have limited capacity for compensatory feeding. While metabolic responses to temperature stress have been investigated in some other copepods such as *Acartia tonsa* (Nielsen *et al.*, 2007), metabolic studies remain scarce for the early life stages and are particularly lacking under ecologically realistic heatwave conditions in *C. finmarchicus*.

Here, we quantify metabolic rates alongside life-history responses in early life stages of *C. finmarchicus* under heat stress and food availability. We measure resting metabolic rate, hatching success, developmental rate, body size and survival to establish a mechanistic link between metabolic demand and performance. We hypothesize that elevated temperatures will accelerate development but increase mortality, particularly under food limitation, and that metabolic rate will increase with temperature, leading to faster depletion of energy reserves and reduced survival under heat stress.

## MATERIALS AND METHODS

### Biological model

The *C. finmarchicus* culture used in this study was first established in 2004 from stage V copepodites collected in Trondheimfjorden using a Nansen zooplankton net (70 cm Ø, 180 μm mesh, Hydro-Bios, Kiel, Germany) during vertical hauls (Hansen *et al.*, 2007). Since then, the culture has been maintained at NTNU SeaLab (Trondheim) in 280 L polyester containers with natural seawater at 10°C ± 2°C and ambient salinity. The copepods are fed a mixture of the unicellular algae *Rhodomonas baltica* and *Dunaliella tertiolecta*, with algal carbon concentrations exceeding 150 μg C L^−1^ to support optimal growth and development (Campbell, 2006). In the wild, *C. finmarchicus* typically has a 1-year life cycle. Females release their eggs close to the surface of the water, where phytoplankton production is sufficient to support both females and hatched nauplii, with egg production typically increasing in spring as algal densities rise. Upon hatching, individuals go through six naupliar stages (N1 to N6) and five copepodite stages (C1 to C5) before reaching maturity as adults (C6). The N3 stage is particularly important, as it marks the onset of feeding, which is critical for continued development (Jung-Madsen and Nielsen, 2015).

Eggs were collected from 250 cultured *C. finmarchicus* females placed in a 45 L tank and allowed to shed eggs for 12 h. Following incubation, the water volume was gently reduced to 3 L by siphoning through a 64 μm mesh to retain eggs while removing smaller particles. The concentrated sample was then passed over a 300 μm sieve to separate and remove adult females from the eggs. Eggs were subsequently rinsed to remove algae and fecal pellets, and subsampled aliquots were used for the experiments. All handling was conducted at 10°C to minimize stress. In all experiments, egg densities were kept low to minimize potential density-dependent effects on development and metabolism.

### Experiment 1: effect of temperature on hatching success

**Fig. 1:**
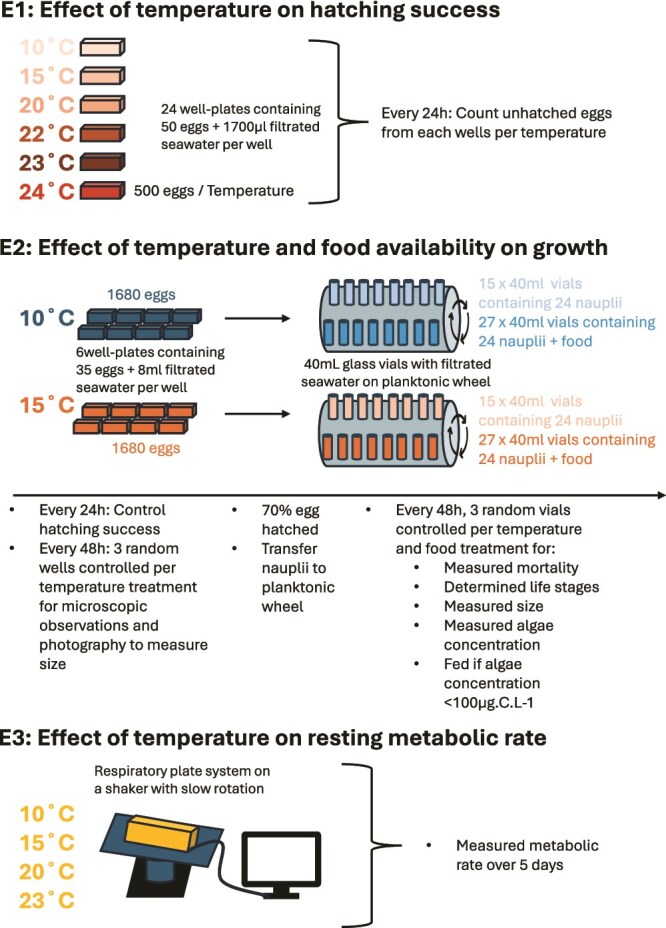
Summary of the 3 experiments. Temperature is in degrees Celsius.

To investigate the effect of temperature on the hatching success of *C. finmarchicus*, we incubated fertilized eggs in 24-well plates (VWR® Multiwell Cell Culture Plates) at each of the following temperatures: 10°C, 15°C, 20°C, 22°C, 23°C and 24°C (±1°C).On each plate, 10 wells were filled with 50 eggs and 1700 μL of sterile filtered, oxygenated natural seawater (Fig. 1), resulting in a total of *N* = 500 eggs per temperature treatment. Each well was treated as an independent replicate. Temperature was recorded every 10 min using a Testo 174t thermometer. As *C. finmarchicus* begins feeding at the N3 stage (Jung-Madsen and Nielsen, 2015), no food was added to preserve water quality. Hatching success was assessed every 24 h by visually inspecting the 10 wells. The number of unhatched eggs was counted using an inverted light microscope (Nikon eclipse TS100 at ×400) equipped with a microscope camera (Leica MC170HD with ×1 relay tube), and the number of hatched eggs was inferred accordingly. Observations continued until the hatching success reached a plateau.

### Experiment 2: effect of temperature and food availability on growth

To investigate the effect of temperature and food availability on the growth of *C. finmarchicus*, two identical climate-controlled rooms were set to 10°C ± 1°C and 15°C ± 1°C, respectively. For each temperature condition, eight 6-well plates (VWR® Multiwell Cell Culture Plates) were prepared, each containing 35 eggs and 8 mL of sterile-filtered natural seawater in equilibrium with atmospheric air, resulting in a total of *N* = 1680 eggs per temperature (Fig. 1). Egg densities in Experiment 2 differed slightly from Experiments 1 and 3 to allow for increased replication and to facilitate accurate handling and counting of individuals overtime. Hatching success was assessed in each well as described in Experiment 1. Once ~70% of the eggs had hatched, 24 nauplii from each well were individually transferred using a pipette into 40 mL glass vials filled with filtered and aerated seawater (Fig. 1). For each temperature, 27 vials were supplemented with *R. baltica* in *ad libitum* quantities (~500 μg C L^−1^), while 15 vials were left without *R. baltica* as the starvation treatment. The vials were evenly distributed on two planktonic wheels rotating at 0.8 rpm in each climate chamber, ensuring that algal particles remained in suspension and accessible to the nauplii. Every 48 h, three vials per temperature and food treatment were randomly sampled to monitor naupliar development, mortality and algal concentration. Sampled vials were not re-used to avoid handling bias. Seawater from each sampled vial was filtered through a 100-μm cell strainer (Corning Falcon®) to recover the nauplii and to measure algal concentration using a particle counter (Coulter Multisizer 3). Algal carbon concentration was estimated from particle counts to ensure food availability remained above the critical threshold of ~100 μg C L^−1^, below which *C. finmarchicus* development can be impaired (Campbell *et al.*, 2001). When concentrations dropped below this level, all nauplii from the given temperature treatment were transferred to fresh, aerated, filtered seawater containing 500 μg C L^−1^ of *R. baltica*, which also helped prevent hypoxia from algal respiration. Recovered nauplii from the three sampled vials were rinsed and individually transferred with a pipette into seawater drops for observation. Individuals immobile for several seconds and/or showing signs of decomposition were recorded as dead. For development and size measurement, each nauplius present in the containers was anesthetized by adding two drops of Finquel® anesthetic (Trikainmesilat) and photographed through an inverted light microscope (Nikon eclipse TS100 at ×400) with a microscope camera (Leica MC170HD with ×1 relay tube). The stages of development were determined partly based on the posterior end of the body and distal segment of the first antennae as done in previous studies (Ogilvie, 1956; Hygum *et al.*, 2000). Each alive nauplius per vial was then assigned a life stage. Five individuals of the majority stage development were measured per replicate. The length, width and area of the prosome were measured with ImageJ version1.43 software (Schneider *et al.*, 2012). The experiment ended when all the wells were sampled to assess mortality and growth.

### Experiment 3: effect of temperature on resting metabolic rate of eggs and nauplii

To assess the effect of temperature on metabolic rate, we used a multi-channel microplate respirometry system (Loligo® Systems, Denmark) to measure oxygen consumption in *C. finmarchicus* eggs over 5 days of temperature exposure. For each temperature (10, 15, 20 and 23 ± 1°C), 10 wells of a 24-well microplate were filled with 50 eggs and 1700 μL of filtered, oxygenated seawater (Fig. 1). The plate was placed on a slow-rotating shaker to prevent eggs from blocking the sensors. The positions of the 10 sample wells and 6 blanks were randomized. Oxygen concentration (mg/L) was monitored every 10 min at each temperature (Supplementary Fig. S1). Metabolic rate was estimated from the rate of decline in oxygen concentration over time after 9 h for eggs and 42 h for nauplii. Calibration and oxygen calculations followed the procedure described in Rousseau *et al.* (2025). Although body size can affect metabolic rate (Gillooly *et al.*, 2001), size correction was not applied due to the minimal size variation among eggs (0.13 ± 0.004 mm, *N* = 10).

### Statistics

All statistical analyses were performed in RStudio v4.2.3 (R Core Team, 2023). Generalized or linear mixed-effects models were used depending on the data structure (Supplementary Table S1). Fixed effects included temperature, day, developmental stage and food availability, with “well” treated as a random factor where repeated measures occurred. Appropriate error distributions (Poisson or Gaussian) were applied according to data type. Post hoc comparisons were conducted using the Tukey–Kramer method (*emmeans* package (Lenth, 2025)). Model assumptions were verified through standard diagnostic plots to assess residual normality, homoscedasticity and influence.

## RESULTS

### Increasing temperature led to reduced hatching success

**Fig. 2:**
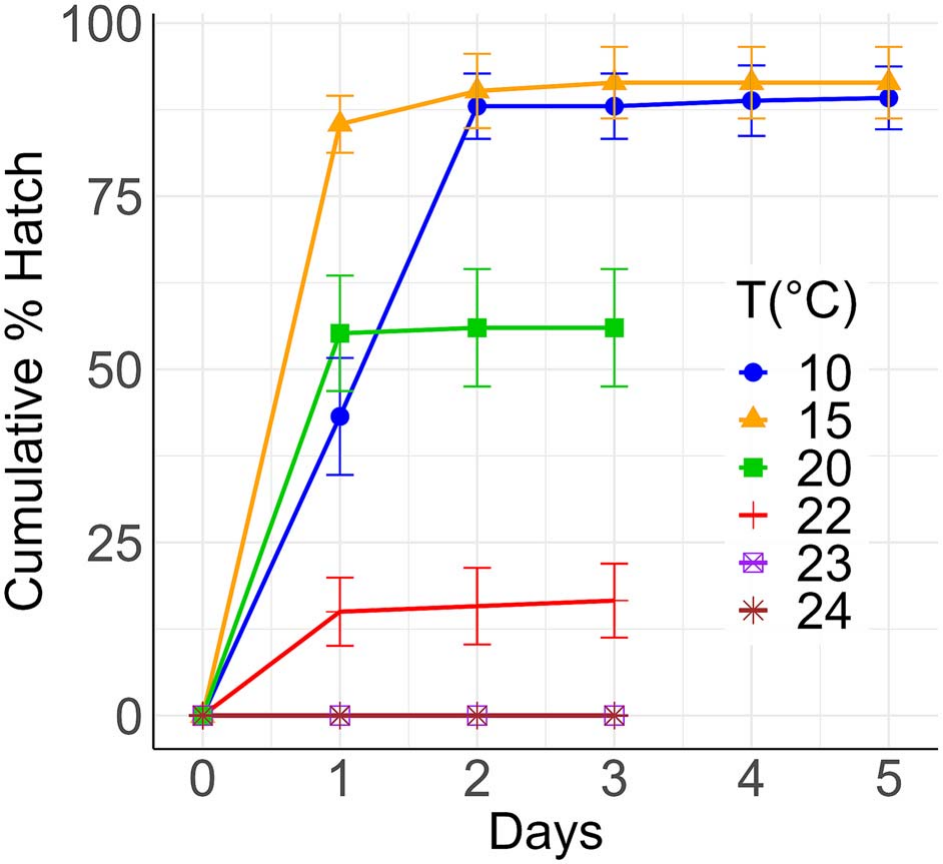
Cumulative percentage of eggs hatched over time at different temperatures (°C). T: Temperature, is degree Celsius. *N* = 60 wells, 10 wells containing 50 eggs for each temperature treatment. Data are presented as cumulative percentage hatch over time for visualization, while statistical analyses were performed on the original daily count data.

Hatching success depended on temperature and time, with a significant Temperature × Day interaction (Estimate = −0.231, *P* < 0.01; Fig. 2, Supplementary Table S2), indicating that temperature affected the timing of hatching. At 15°C, ~90% of eggs hatched within the first day, whereas at 10°C, a similar level of hatching was only reached after 2 days. Total hatching success decreased at higher temperatures, with ~55% hatching at 20°C and ~15% at 24°C, while no hatching occurred at 23°C and 24°C.

### Development time and growth

**Fig. 3:**
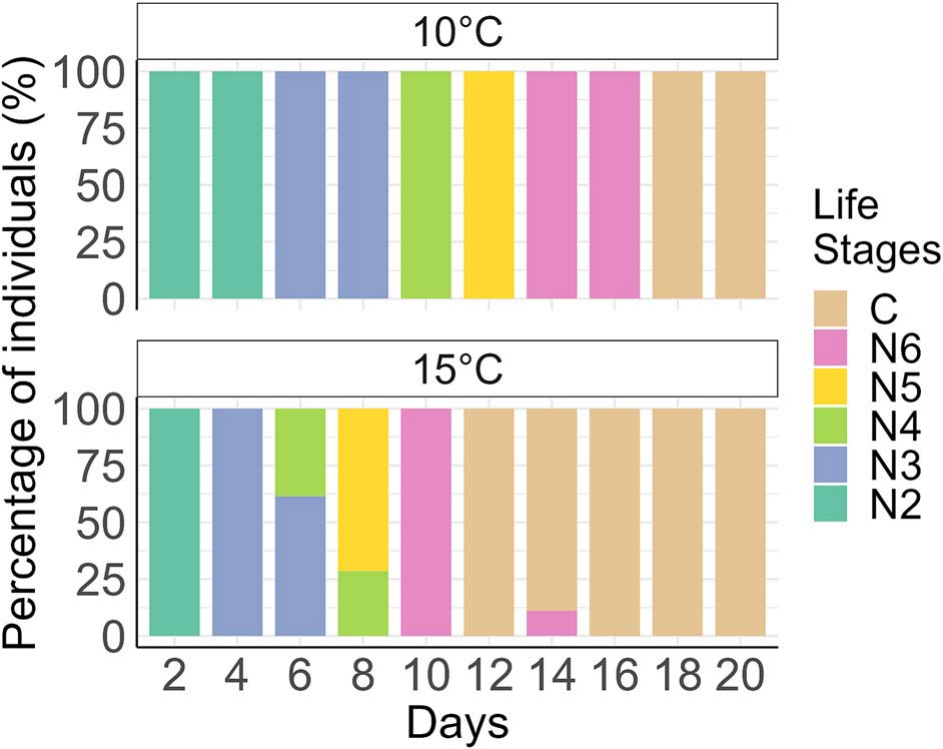
Percentage of individual’s life stages over time depending on temperature. Temperature is degree, Celsius. Life stages are reported as N: nauplii and C: copepodite. *N* = 320 individuals, 159 at 10°C and 161, at 15°C. Data are presented as percentage of individuals for visualization, while statistical analyses were performed on the original daily count data.

There was a marginally significant three-way interaction between temperature, day and developmental stage (*F*₂,₃ = 2.49, *P* = 0.083; Fig. 3, Supplementary Table S3), with a trend suggesting that temperature influenced the rate of progression through naupliar stages over time. The development through naupliar stages (N2–N6) appeared faster at 15°C than at 10°C (Fig. 3). The significant day by developmental stage interaction (*F*_4,5_ = 3.65, *P* < 0.01, Supplementary Table S3) further indicated that developmental progression diverged over time, with individuals at 15°C advancing faster through naupliar stages than at 10°C, leading to an increasing lag in stage composition at lower temperature. No significant interactions were found between temperature and days or between temperature and developmental stage (both *P* > 0.05, Supplementary Table S3).

**Fig. 4:**
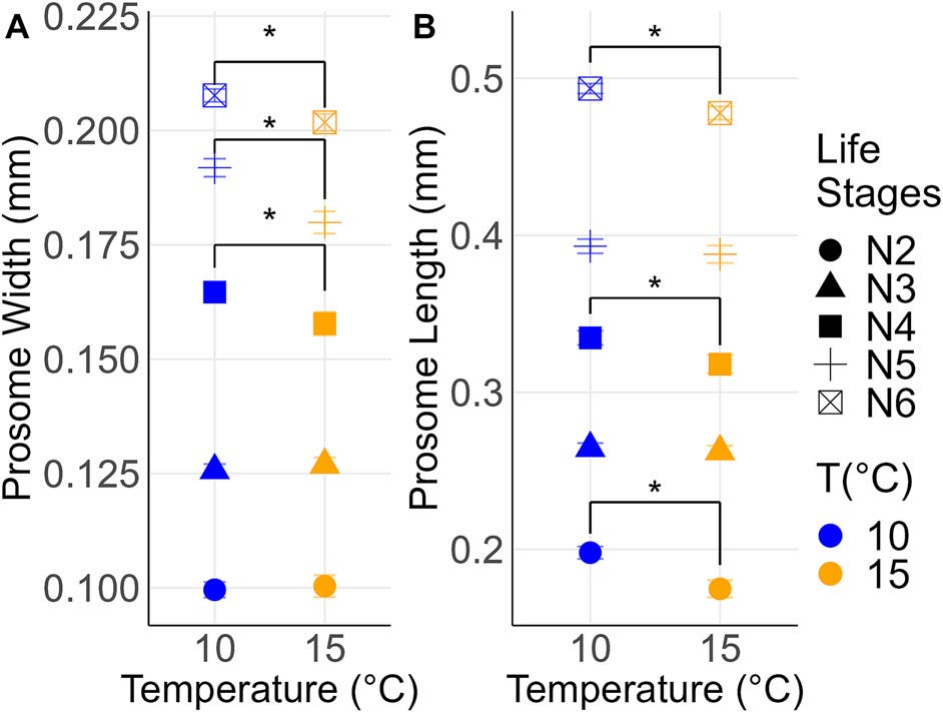
Prosome width (**A**) and length (**B**) of different copepod life stages at 10°C and 15°C. Temperature is degree Celsius. Life stages are reported as N: nauplii. Measurements of prosome length and width are in millimeters. *N* = 179 individuals, 110 at 10°C and 69 at 15°C.

Although copepods reared at 15°C developed more rapidly, they attained smaller body sizes at the same life stage compared to those at 10°C. A significant interaction between temperature and developmental stage was detected for prosome width (*F*_4,169_ = 4.13, *P* < 0.01, Supplementary Table S4, Fig. 4A), whereas a marginally significant interaction was observed for prosome length (*F*_4,169_ = 2.13, *P* = 0.07, Supplementary Table S5, Fig. 4B). Prosome length was significantly shorter at 15°C than at 10°C at the N2, N4 and N6 stages (*P* < 0.01 in all cases), but no significant differences were found for the N3 or N5 stages (*P* > 0.1 in all cases). Similarly, prosome width was greater at 10°C than at 15°C at the N4, N5 and N6 stages (*P* < 0.01 in all cases), with no significant difference observed for N3 stage (*P* > 0.1 in all cases).

### Metabolic rate

**Fig. 5:**
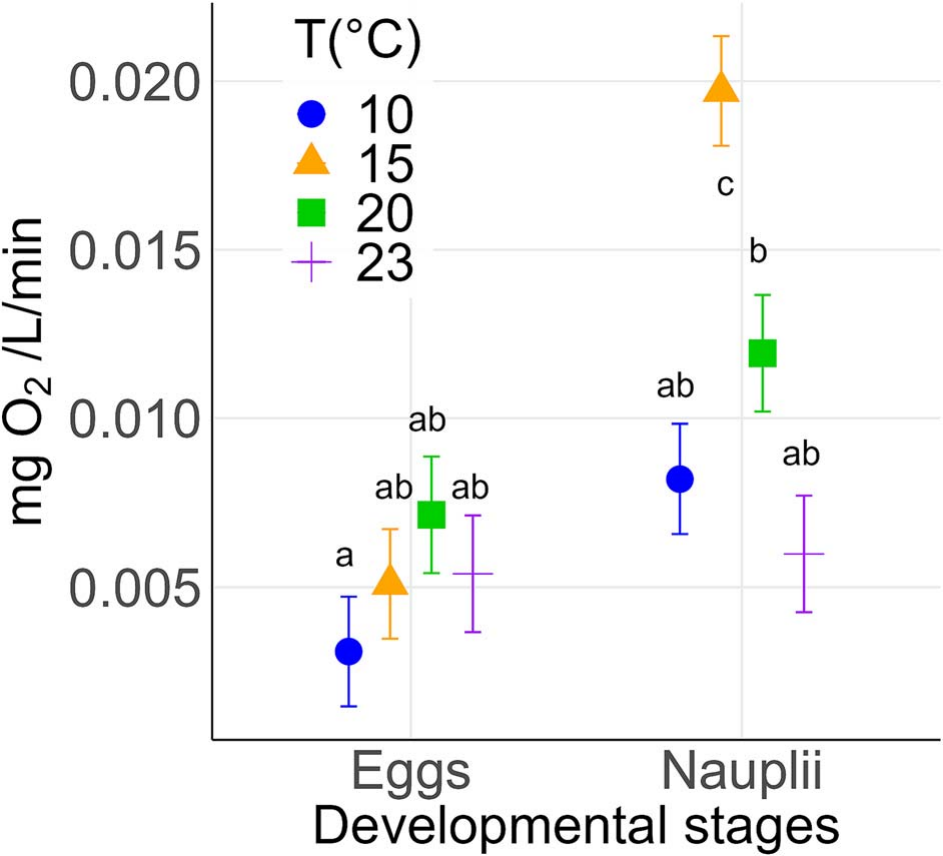
Metabolic rate of eggs and nauplii at different temperatures (°C). Metabolic rate was measured in wells containing 50 eggs and is expressed as mg/L of oxygen consumed per minute. Sample sizes: *N* = 34 wells (nine wells for 10°C and 15°C; eight wells for 20°C and 23°C).

Oxygen concentration declined over time in all temperature treatments, with steeper declines observed at higher temperatures (Supplementary Fig. S1). There was a significant interaction between temperature and life stage on oxygen consumption (*F*_3,44.77_ = 6.76, *P* < 0.01, Fig. 5, Supplementary Table S6). There was no significant effect of temperature on eggs; however, oxygen consumption of nauplii at 15°C was significantly higher than at 10°C, 20°C and 23°C (*P* < 0.01 in all cases).

### Mortality

**Fig. 6:**
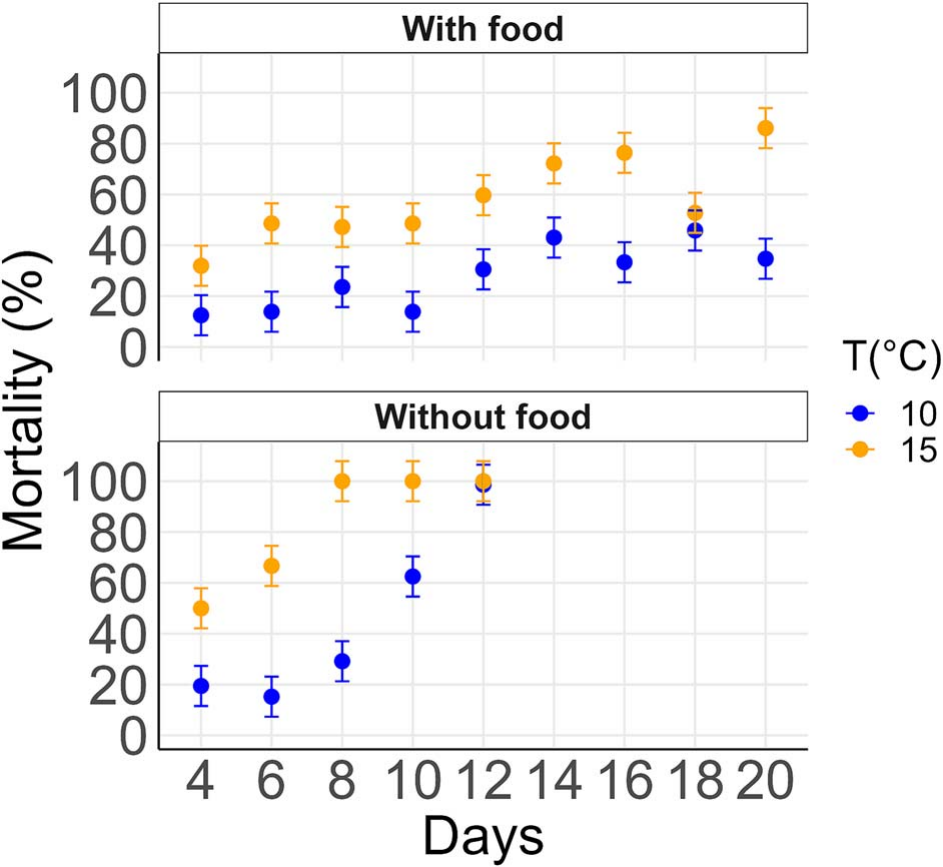
Percentage of nauplii mortality overtime depending on temperature. Temperature is degree Celsius. *N* = 84 vials containing 25 nauplii. Forty-two vials for each temperature.

A significant three-way interaction between temperature, time and food availability was detected for naupliar mortality (*F*_4,56_ = 2.92, *P* = 0.02, Fig. 6, Supplementary Table S7). Mortality increased with both temperature and time, while food availability reduced mortality across treatments. In the absence of food, nauplii reached 100% mortality by Day 8 at 15°C and by Day 12 at 10°C. In contrast, when food was available, surviving individuals were still observed on Day 20 at both temperatures. Post hoc comparisons confirmed that mortality was significantly higher without food at 15°C than at 10°C on Day 4 (*T*_56_ = −2.74, *P* = 0.04), Day 6 (*T*_56_ = −4.61, *P* < 0.01), Day 8 (*T*_56_ = −6.35, *P* < 0.01) and Day 10(*T*_56_ = −3.36, *P* < 0.01), whereas on Day 12, both temperature treatments reached 100% mortality. Differences in survival between temperatures remained evident under food-supplied conditions at Day 6, Day 10, Day 16 and Day 20 (*P* < 0.01 in all cases).

## DISCUSSION

We observed a pronounced effect of temperature on hatching success, with significantly faster hatching success at 15°C compared to 10°C, but no hatching at 23°C and 24°C. Similar patterns have been reported in other studies on *C. finmarchicus*. Preziosi and Runge (2014) found that hatching success remained stable between 6°C and 19°C, with no viable eggs produced at 23°C, while Weydmann *et al.* (2015) observed shorter egg development time from 0°C to 10°C without major changes in hatching success within that lower thermal range. Taken together, these studies indicate that *C. finmarchicus* eggs tolerate moderate warming but significantly decreased hatching success above ~20°C, which is consistent with our findings. Temperature-dependent responses have long been documented in *Calanus* spp., particularly in *C. finmarchicus* and *Calanus glacialis* (Hirche, 1989, 1990; Runge and Plourde, 1996; Hirche *et al.*, 1997). However, the magnitude and nature of temperature sensitivity can vary among congeneric species. For example, in *Calanus helgolandicus*, hatching success remained high even at 22.5°C but with accelerated development (Laabir *et al.*, 1995). At high temperatures, egg viability may decline due to biochemical or structural degradation. Heat exposure can reduce the fatty acid and cholesterol content in egg membranes (Weaver *et al.*, 2008) and cause membrane rupture in copepod eggs (Jiang *et al.*, 2021), likely explaining the hatching failure at 23°C and 24°C in our study. Warming can shift hatching and developmental timing, altering phenology and potentially reducing offspring quality and growth (Walther *et al.*, 2002).

Naupliar development was faster at 15°C, with individuals reaching more advanced life stages compared to those at 10°C within the same time period. However, this accelerated development came with clear trade-offs where nauplii at 15°C had significantly shorter prosome size than those at 10°C, consistent with the temperature-size rule, where ectoderms grow faster but reach smaller sizes at higher temperatures (Atkinson, 1994). Similar temperature-dependent development rates have been documented in *C. finmarchicus* across multiple thermal ranges (4°C–12°C: Campbell *et al.*, 2001; 0°C–10°C: Jung-Madsen and Nielsen, 2015), as well as in other copepods such as *C. helgolandicus* (Laabir *et al.*, 1995), *Acartia* spp. (Garzke *et al.*, 2015; Jo *et al.*, 2019) and *Tachidius discipes* (Vigliano Relva *et al.*, 2025).

In *C. finmarchicus*, Campbell *et al.* (2001) found temperature-related size differences at the N6 stage but not the N3 stage, which aligns with our results showing size reduction in all naupliar stages except N3. This stage is the first feeding stage and may represent a developmental threshold requiring a minimum size before initiating feeding. Furthermore, Jung-Madsen and Nielsen reported stage-specific responses to temperature in *C. finmarchicus*, where the durations of early naupliar stages (N1–N2) were shorter but the N3 stage was longer at higher temperatures, indicating nonlinear developmental responses. Our results showing a widening developmental gap between 10°C and 15°C over time support this idea and suggest that small early differences can amplify across stages. Although copepodite stages were not measured in our study due to technical constraints, we expect that size differences would become even more pronounced in later stages, following the pattern described by Forster *et al.* (2011).

The observed reduction in naupliar body size under warming is consistent with our metabolic observations. Smaller body size at higher temperatures likely reflects increased metabolic demands, reducing the energy available for growth (Forster *et al.*, 2012; Sokolova *et al.*, 2012; Schulte, 2015). While we observed no significant effect of temperature on egg metabolic rates, naupliar oxygen consumption was significantly higher at 15°C than at 10°C, indicating increased energetic demand after hatching. In contrast, Nielsen *et al.* (2007) reported an exponential increase in egg oxygen consumption with temperature in *A. tonsa*. This difference may reflect species-specific responses, methodological differences or the relatively low metabolic activity of eggs compared to actively developing nauplii. It is also possible that temperature effects on metabolism become more pronounced after hatching, when organisms transition from relying solely on yolk reserves to more energetically demanding developmental processes. In our study, respiration rates were not normalized to body size and thus reflect the combined effects of temperature and developmental stage. As individuals were not fed, biomass could only decrease over time due to metabolic expenditure, with higher temperatures likely accelerating this loss. Consequently, part of the observed variation in metabolic rate may reflect temperature-driven differences in biomass rather than temperature alone. Together, these findings provide novel insight into early life stage physiology in *C. finmarchicus* and support the hypothesis that temperature-driven increases in metabolic demand contribute to reduced naupliar body size under warming.

Mortality increased with both temperature and time, while food availability partly mitigated this effect. At 15°C, nauplii survived for shorter periods than at 10°C across regardless of food availability, indicating that elevated temperature reduces the window of survival during early development. Notably, under starvation conditions, nauplii at 10°C exhibited similar survival to fed individuals for several days, whereas this buffering capacity was reduced at 15°C. This suggests that higher temperatures increase sensitivity to food limitation by accelerating metabolic demand. Earlier hatching pattern has important implications under marine heatwave conditions, which can last from days to several weeks (Hobday *et al.*, 2016). If nauplii hatch during such events, increased metabolic demand combined with reduced starvation tolerance may amplify the consequences of a mismatch with phytoplankton blooms, thereby lowering the probability of surviving to later developmental stages.

At a broader ecological scale, our results suggest multiple pathways through which warming can affect copepod populations and general marine ecosystems. Increased metabolic demand under higher temperatures as observed in our study may lead to reduced naupliar size, which in turn can limit lipid storage both directly through allometric constraints and indirectly via reduced dietary carbon assimilation and lipid biosynthesis (Sahota *et al.*, 2022). Consequently, smaller and less lipid-rich nauplii may provide lower nutritional value to predators, ultimately reducing trophic transfer efficiency and the capacity for carbon and nutrient transport through the food web (Steinberg and Landry, 2017). At the same time, accelerated development may shift the timing of naupliar stages, increasing the risk of phenological mismatch with phytoplankton blooms and/or creating a trophic mismatch with predators (Ji *et al.*, 2010; Forsblom *et al.*, 2024). Earlier hatching combined with faster development could result in peak feeding demand occurring before maximum food availability, further exacerbating mortality and limiting growth.

## CONCLUSIONS

Overall, our results demonstrate that temperature exerts strong, stage-specific effects on early development in *C. finmarchicus*. Moderate warming (15°C) accelerated development but reduced body size and survival, revealing physiological trade-offs between growth and fitness. By combining metabolic and developmental data, our results suggest that increased energetic demand at higher temperatures contributes to the reduced body size and survival observed in nauplii. Above 20°C, hatching success declined sharply, highlighting the high thermal sensitivity of early stages. Although food availability partially mitigated these effects, it did not offset the energetic costs of elevated temperatures, underscoring the narrow thermal window for successful development in this species under heatwave conditions. These findings suggest that ocean warming may favor smaller, shorter-lived individuals, potentially reducing population productivity and altering trophic dynamics in North Atlantic ecosystems.

## Supplementary Material

V2_Supplement_material___Metabolic_and_life_history_effects_of_heat_in_copepods_fbag048

## Data Availability

https://doi.org/10.5281/zenodo.17569581
