# Supplementary material for: Metabolic and life-history effects of heat stress in early life stages of a marine copepod, Calanus finmarchicus
Source: J Plankton Res. 2026 Jun 30;48(4):fbag048. doi: 10.1093/plankt/fbag048 (PMC13317939; doi:10.1093/plankt/fbag048)
Supplement: V2_Supplement_material___Metabolic_and_life_history_effects_of_heat_in_copepods_fbag048 [file v2_supplement_material___metabolic_and_life_history_effects_of_heat_in_copepods_fbag048.docx]

**Supplement material -** **Metabolic and life-history effects of heat stress in early life stages of a marine copepod, *Calanus finmarchicus*.**

**
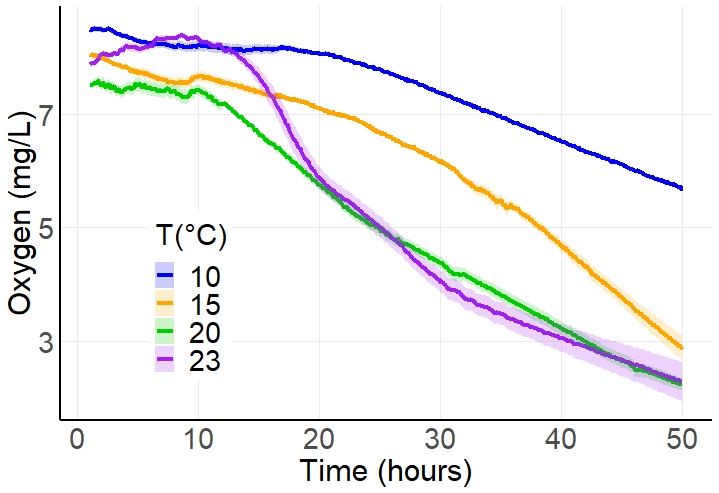
**

**Figure S1. Oxygen levels overtime depending on temperatures (˚C).** Lines represent mean oxygen concentrations, and shaded areas indicate ± standard error. Oxygen levels were measured in wells containing 50 eggs and are expressed as mg L⁻¹. Sample sizes were N = 34 wells in total (n = 9 wells at 10 °C and 15 °C; n = 8 wells at 20 °C and 23 °C).

**Table S1. Summary of statistical models used to analyse *Calanus finmarchicus* developmental and physiological responses.**

| **Response variable** | **Model type** | **Distribution** | **Fixed effects** | **Random effects** | **Notes** |
| --- | --- | --- | --- | --- | --- |
| Hatching | GLMM | Poisson | Temperature, days | Well | Repeated counts |
| Developmental stage | GLM | Poisson | Temperature, day, life stage | / | Count data |
| Prosome length & width | GLM | Gaussian | Temperature, day | / | Continuous variable |
| Mortality | GLM | Gaussian | Temperature, day, food | / | % mortality per well |
| Metabolic rate | LMM | Gaussian | Temperature, life stage | Well | Continuous variable |

**Table S2. ANOVA analysis of hatching**. *N*=60 wells, 10 wells containing 50 eggs for each temperature treatment resulting to 500 eggs per temperature treatment. Signification codes: ‘***’ p < 0.001; ‘**’ p < 0.01; ‘*’ p < 0.05; ‘.’ p < 0.1; ‘ ’ p ≥ 0.1 (not significant).

| **Effect** | **Estimate** | **Std. Error** | **Z value** | **Pr (>\|z\|)** |
| --- | --- | --- | --- | --- |
| **(Intercept)** | 2.710 | 0.067 | 40.297 | < 2e-16 *** |
| **Temperature 15** | 0.325 | 0.091 | 3.582 | 0.00034 *** |
| **Temperature20** | -0.255 | 0.107 | -2.384 | 0.01713 * |
| **Temperature22** | -1.596 | 0.174 | -9.193 | < 2e-16 *** |
| **Temperature23** | -34.530 | 2.495e+06 | 0.000 | 0.99999 |
| **Temperature24** | -31.900 | 6.796e+05 | 0.000 | 0.99996 |
| **Days** | -0.351 | 0.031 | -11.366 | < 2e-16 *** |
| **Temperature15 × Days** | -0.231 | 0.047 | -4.881 | 1.06e-06 *** |
| **Temperature20 × Days** | -0.056 | 0.065 | -0.856 | 0.39178 |
| **Temperature22 × Days** | 0.060 | 0.106 | 0.562 | 0.57436 |
| **Temperature23 × Days** | -0.886 | 3.204e+06 | 0.000 | 1.00000 |
| **Temperature24 × Days** | -1.387 | 1.268e+06 | 0.000 | 1.00000 |

**Table S3. ANOVA analyses Temperature, Days and Developmental Stages on individuals count**. *N*=320 individuals, 159 at 10˚C and 161 at 15 ˚C. Signification codes: ‘***’ p < 0.001; ‘**’ p < 0.01; ‘*’ p < 0.05; ‘.’ p < 0.1; ‘ ’ p ≥ 0.1 (not significant).

| **Parameters** | **Df** | **Df** | **F value** | **P value** |
| --- | --- | --- | --- | --- |
| **Temperature** | 1 | 21 | 4.9789 | 0.02566 * |
| **Days** | 1 | 20 | 23.2280 | 1.439e-06 *** |
| **Developmental stage** | 5 | 15 | 2.8553 | 0.01395 * |
| **Temperature x Days** | 1 | 14 | 0.0276 | 0.86801 |
| **Temperature x Dev. stage** | 5 | 9 | 1.2170 | 0.29804 |
| **Day x Dev. stage** | 4 | 5 | 3.6517 | 0.00559 ** |
| **Temperature x Day x Dev. stage** | 2 | 3 | 2.4904 | 0.08288 . |

**Table S4. Anova analyses of effect of temperature and developmental stage on prosome width**. *N*=179, 110 at 10 ˚C and 69 at 15 ˚C. Signification codes: ‘***’ p < 0.001; ‘**’ p < 0.01; ‘*’ p < 0.05; ‘.’ p < 0.1; ‘ ’ p ≥ 0.1 (not significant).

| **Prosome Width** |  |  |  |  |
| --- | --- | --- | --- | --- |
| **Parameters** | **Df** | **Df** | **F value** | **P value** |
| **Temperature** | 1 | 177 | 14.2092 | 0.0002258 *** |
| **Developmental stage** | 4 | 173 | 1203.1040 | < 2.2e-16 *** |
| **Temperature x Dev. stage** | 4 | 169 | 4.1399 | 0.0031702 ** |

**Table S5. Anova analyses of effect of temperature and developmental stage on prosome length**. *N*=179, 110 at 10 ˚C and 69 at 15 ˚C. Signification codes: ‘***’ p < 0.001; ‘**’ p < 0.01; ‘*’ p < 0.05; ‘.’ p < 0.1; ‘ ’ p ≥ 0.1 (not significant**).**

| **Prosome Length** |  |  |  |  |
| --- | --- | --- | --- | --- |
| **Parameters** | **Df** | **Df** | **F value** | **P value** |
| **Temperature** | 1 | 177 | 25.872 | 9.621e-07 *** |
| **Developmental stage** | 4 | 173 | 1684.512 | < 2.2e-16 *** |
| **Temperature x Dev. stage** | 4 | 169 | 2.134 | 0.07873 . |

**Table S6. Anova analyses of the effect of temperature on eggs and nauplii metabolic rates**. *N*= 34 wells, 9 wells for 10˚C and 15˚C, 8 wells for 20˚C and 23˚C. Signification codes: ‘***’ p < 0.001; ‘**’ p < 0.01; ‘*’ p < 0.05; ‘.’ p < 0.1; ‘ ’ p ≥ 0.1 (not significant).

| **Parameters** | **Sum sq** | **Df** | **Df** | **F value** | **P value** |
| --- | --- | --- | --- | --- | --- |
| **Temperature** | 0.00054706 | 3 | 59.344 | 8.1133 | 0.0001298 *** |
| **Developmental stage** | 0.00066626 | 1 | 44.804 | 29.6430 | 2.091e-06 *** |
| **Temperature x Dev. stage** | 0.00045640 | 3 | 44.771 | 6.7688 | 0.0007325 *** |

**Table S7. Anova analyses the effects of time, temperature and food on mortality**. Mortality binomial data have been converted into percentage. *N*= 84 vials containing 25 nauplii. 42 vials for each temperature. Signification codes: ‘***’ p < 0.001; ‘**’ p < 0.01; ‘*’ p < 0.05; ‘.’ p < 0.1; ‘ ’ p ≥ 0.1 (not significant).

| **Parameters** | **Df** | **Df** | **F value** | **P value** |
| --- | --- | --- | --- | --- |
| **Days** | 8 | 75 | 10.3052 | 1.005e-08 *** |
| **Temperature** | 1 | 74 | 123.5249 | 8.660e-16 *** |
| **Food** | 1 | 73 | 77.7368 | 3.570e-12 *** |
| **Days x Temperature** | 8 | 65 | 2.5268 | 0.0201520 * |
| **Days x Food** | 4 | 61 | 6.8179 | 0.0001523 *** |
| **Temperature x Food** | 1 | 60 | 2.0131 | 0.1614931 |
| **Days x Temperature x Food** | 4 | 56 | 2.9237 | 0.0288336 * |
